# Advanced driving assistance integration in electric motorcycles: road surface classification with a focus on gravel detection using deep learning

**DOI:** 10.3389/frai.2025.1520557

**Published:** 2025-02-14

**Authors:** Ranan Venancio, Vitor Filipe, Adelaide Cerveira, Lio Gonçalves

**Affiliations:** ^1^School of Science and Engineering, University of Trás-os-Montes and Alto Douro, Vila Real, Portugal; ^2^Institute for Systems and Computer Engineering - Technology and Science (INESC TEC), Porto, Portugal

**Keywords:** advanced driving assistance, electric motorcycles, road surface classification, deep learning, gravel detection

## Abstract

Riding a motorcycle involves risks that can be minimized through advanced sensing and response systems to assist the rider. The use of camera-collected images to monitor road conditions can aid in the development of tools designed to enhance rider safety and prevent accidents. This paper proposes a method for developing deep learning models designed to operate efficiently on embedded systems like the Raspberry Pi, facilitating real-time decisions that consider the road condition. Our research tests and compares several state-of-the-art convolutional neural network architectures, including EfficientNet and Inception, to determine which offers the best balance between inference time and accuracy. Specifically, we measured top-1 accuracy and inference time on a Raspberry Pi, identifying EfficientNetV2 as the most suitable model due to its optimal trade-off between performance and computational demand. The model's top-1 accuracy significantly outperformed other models while maintaining competitive inference speeds, making it ideal for real-time applications in traffic-dense urban settings.

## 1 Introduction

Integrating advanced safety features in urban mobility solutions, particularly within the context of electric motorcycles, is critical for fostering efficient, comfortable, and environmentally sustainable urban transportation. The project “A-MoVeR—Mobilizing Agenda for the Development of Products & Systems toward an Intelligent and Green Mobility” addresses this challenge by promoting advancements in green mobility solutions. A pivotal goal of this agenda is the development of a new electric motorcycle with extended autonomy tailored for urban environments. This motorcycle seeks to minimize emissions and incorporates intelligent systems to enhance rider safety and comfort.

Safety in urban mobility involves diverse technologies designed to identify and adapt to dynamic environmental conditions. These systems encompass collision avoidance, pedestrian detection, traffic sign recognition, and evaluating road surface conditions, including detecting hazardous materials like gravel and assessing asphalt quality. These features are indispensable in densely populated urban settings, where diverse road conditions and traffic scenarios complicate rider safety. The complexity of urban mobility underscores the necessity for advanced road surface analysis technologies, a focal point of this study.

The significance of road surface analysis has been well-established in the literature. For example, the dataset introduced by Zhao and Wei (2022) provides detailed annotations of road surface images, enabling the development and evaluation of machine learning models for road condition assessment. This dataset encompasses a diverse range of surface types and conditions, organized into 27 classes based on material (e.g., asphalt, concrete, gravel), friction (e.g., dry, wet, snow, ice), and surface quality (e.g., smooth, slight, severe). Such diversity ensures that machine learning models trained on this dataset are robust to varying road conditions, making it highly relevant for gravel detection, which is critical for motorcycle safety as loose gravel significantly reduces tire traction and control. Building on this, the work of Lee et al. (2021) demonstrates the potential of intelligent sensor systems, such as accelerometers embedded in tires, to classify road surfaces in real time, showcasing the effectiveness of deep learning approaches in such contexts.

A comprehensive review by Botezatu et al. (2024) further underscores the importance of deep learning in road surface analysis. This study highlights state-of-the-art convolutional neural network (CNN) architectures for detection road damage and classifying surfaces based on material and environmental conditions. The review emphasizes the balance between real-time processing and classification accuracy, mainly through innovations like YOLO and hybrid models. These contributions align closely with the objectives of this work, which seeks to address similar challenges in gravel detection for electric motorcycles.

While significant progress has been made in road damage detection and surface classification, as evidenced by the studies above, gaps remain in the practical implementation of these technologies for motorcycles. To the best of our knowledge, this study represents the first effort to develop a driving assistance framework tailored explicitly for motorcycles, addressing critical safety concerns in urban mobility. By leveraging the “Road Surface Image Dataset with Detailed Annotations” (Zhao and Wei, 2022), we implemented and validated deep learning models on an embedded system, the Raspberry Pi, as a prototype for eventual integration into a test motorcycle. To address the challenge of class imbalance in the dataset, which includes rare but hazardous conditions such as gravel or ice, we employed Focal Loss (Lin et al., 2018), a technique that enhances model performance by focusing on underrepresented classes. This approach focuses on selecting models, such as ConvNeXt, EfficientNet, and Inception, that balance performance and inference time, ensuring feasibility for real-time applications. Unlike prior studies, this work uniquely targets the enhancement of motorcyclist safety in urban environments, offering a novel contribution to the field.

The remainder of this paper is structured as follows. Section 2.1 covers the data collection, preprocessing, and augmentation methods employed, along with the categorization and labeling processes for different road surface conditions, which are vital for training effective deep learning models. Then, Section 2.2 introduces the deep learning architectures evaluated in this study, including EfficientNetV2, ConvNeXt, and others. Section 2.3 discusses initial training (before refinement) procedures and results, focusing on their potential to detect road surface anomalies such as gravel. Due to the unbalanced nature of the classes in our dataset, this section also discusses the application of fine-tuning and focal loss to improve model performance. In Section 3, the results are presented and analyzed. The evaluation metrics, including top-1 accuracy, inference time, and the effectiveness of focal loss, are analyzed. This section underscores the enhancements from fine-tuning and focal loss with a comparative accuracy and computational efficiency analysis. Section 4 summarizes the main contributions of our research and discusses avenues for future investigations.

## 2 Materials and methods

### 2.1 Dataset

The dataset published by Zhao and Wei (2022) contains ~960 thousand training images, 50 thousand test images, and 20 thousand validation images.

This dataset contains six major classes, consisting of different road surface materials such as *asphalt, concrete, gravel, mud, snow* and *ice*. Asphalt and concrete classes are further sub-categorized within two parameters: humidity and road defect severity, each of which has three categories: *dry, water*, and *wet* for humidity, and *severe, slight, smooth* for road defect severity. As such, asphalt and concrete have nine classes each. *Gravel* and *mud*, on the other hand, are only sub-categorized based on humidity. As such, it only has three sub-classes: *dry, water*, and *wet*. *Snow* is categorized based on whether it is *melted* or *fresh*. *Ice* has no sub-categories. When considering every subclass as independent, this dataset contains 27 classes in total.

To account for different weather conditions such as differences in brightness during the day, data augmentation techniques were utilized by applying transformations to the dataset before training. Such transformations consist of randomly altering image brightness and contrast by 30%, and randomly mirroring the image with a chance of 50%. This process was done dynamically, as part of the data loading routine in the model training scripts.

### 2.2 Model selection

The development of Deep CNNs was significantly influenced by the proliferation of large-scale classification datasets, such as ImageNet (Krizhevsky et al., 2012), which led to the emergence of modern model architectures designed and benchmarked for their performance on datasets that are several orders of magnitude larger than the dataset described in Section 2.1. This and the performance limitations imposed by embedded systems, such as the ones used in Advanced Rider Assistance Systems (ARAS), were considered when selecting the model architectures. To study the impact of embedded system limitations on model inference time, a Raspberry Pi 4 Model B, with 4GB of RAM and a quad-core ARM Cortex-A72 CPU clocked at 1.5Ghz, running Debian GNU/Linux 12 (bookworm) was used to evaluate the performance of the different architectures.

To reduce computational costs both on training and inference, we have decided to select the models that have a relatively high performance-to-inference time trade-off. In particular, the EfficientNet (Tan and Le, 2019), and EfficientNetV2 (Tan and Le, 2021) architectures were designed with this aspect in mind. The MobileNetV3 (Howard et al., 2019) architecture was developed with the primary goal of having small inference speeds and high accuracy when compared to other models of similar size. To compare these highly efficient architectures with more traditional ones, we chose the InceptionV3 (Szegedy et al., 2015) architecture, and we chose ConvNeXt (Liu et al., 2022) for comparison to more recent CNN architectures. The MobileNet architecture (Howard et al., 2017) established the basis for achieving faster inference speeds in CNNs through its use of depth-wise and pointwise convolution filters. Through the replacement of standard convolutional layers by these simpler operations, a reduction of computational cost was achieved while maintaining comparable accuracy to popular models at the time. In the MobileNetV2 architecture (Sandler et al., 2018), this concept was further explored, giving rise to the MBConv block, where a 1 × 1 pointwise convolution filter is followed by a depth-wise 3 × 3 convolution, and finally another 1 × 1 pointwise convolution filter with a linear activation function is used and the result from the previous layer is then added to the inputs for the block. With this block, an even higher efficiency is achieved when compared to the results of the original MobileNet architecture. Thus, the main design principle of these architectures relies mostly on achieving high speed, without greatly compromising accuracy in return, and since such metrics are trivially measured after training, a cost function can be used to estimate the efficiency of a given neural network architecture. This concept was then exploited during MobileNetV3's design, which resulted from optimizing inference speed and accuracy of the MBConv blocks.

The EfficientNet-B0 architecture (Tan and Le, 2019) was designed as a baseline model using Neural Architecture Search (NAS) techniques (Tan et al., 2018) by setting a fixed FLOPs target of 400M and optimizing for both accuracy and FLOPs, which in turn yielded a highly efficient baseline model that uses MobileNet's MBConv block as a base and squeeze-and-excitation optimization (Hu et al., 2018).

Similarly, the EfficientNetV2 was also designed using NAS, but it also reduces the computational costs by using a variant of the previous MBConv block where two of the initial layers are fused into a single operation. The training time was also improved in the EfficientNetV2 architecture by including it in the cost function during the NAS.

After the introduction of the attention mechanism in Transformer architectures (Vaswani et al., 2017) and its applications in the field of Natural Language Processing, the Visual Transformer architecture (Dosovitskiy et al., 2020) explored possible uses for Transformer-based architectures in computer vision, resulting in superior results when compared to the CNN architectures. Using the ResNet architecture (He et al., 2015) as a base architecture and using similar techniques to both the Swin Transformer (Liu et al., 2021) such as layer normalization, as well as inverted residual bottlenecks and depthwise separable convolutions introduced by the MobileNet architectures, the ConvNeXt-T architecture achieves comparable results to that of Vision Transformer architectures while maintaining the simplicity, efficiency, and convenience of well-established CNN networks.

We've chosen the versions of these architectures with the least number of trainable parameters and FLOPs as to prioritize lower inference times. Specifically, the EfficientNet-B0, EfficientNetV2-B0, MobileNetV3-Small, and ConvNeXt-T models were used, with 5.3M, 7.4M, 2.49M, 24M, and 29M trainable parameters, and 0.39B, 0.7B, 0.1B, 5.7B, and 4.5B FLOPs respectively.

To address the risk of overfitting, we have chosen to train these models through transfer learning. To do this, we appended a global average pooling layer to the model, followed by a dense layer, flattened, and then another dense layer.

### 2.3 Training procedures

We have chosen to design, evaluate, and train these models using the Keras framework with TensorFlow as its backend. To reduce training time we have decided to do so on a computer equipped with an NVIDIA^®^ GeForce^®^ RTX 3090 GPU (24GB), an Intel®Core™ i9-12900KF CPU clocked at 3.20GHz, and 32GB of RAM. Training was performed using the Windows Subsystem for Linux 2 feature in Windows 11 to ensure proper use of the aforementioned GPU, as officially recommended by the TensorFlow documentation.

The initial training stage used a Cross-Entropy (CE) loss function, which is defined, based on a single the probability predicted by the model (*p*_*i*_) and its expected value (*y*_*i*_) as:


(1) $$\begin{array}{l} CE\left(p_{i},y_{i}\right)=\{\begin{array}{ll} -\log\left(p_{i}\right) & \text{if }y_{i}=1\\ -\log\left(1-p_{i}\right) & \text{if }y_{i}\neq 1 \end{array} \end{array}$$

For the probabilities on every class, this becomes:


(2) $$\begin{array}{l} CE\left(p,y\right)=\overset{C}{\underset{i=1}{\sum }}CE\left(p_{i},y_{i}\right) \end{array}$$

Where *C* is the amount of classes in the dataset, which in this case is 27, as mentioned in Section 2.1. In the case of transfer learning this suffices, as the main goal is to first train the additional components of the network on the first epochs, and then adapt the pre-trained layers to the new dataset.

Since that the amount of samples for the classes in the dataset varies from approximately four thousand to eighty thousand images, we addressed class imbalance by further training each model for eight more epochs with a focal loss (Lin et al., 2018). This loss function introduces a balancing factor for each class (α_*i*_ ∈ [0, 1]), which can be used to attribute a higher weight for under-represented classes, and a lower weight for over-represented classes. Furthermore, a constant (γ ∈ [0, 5]) is also introduced to modulate the impact of correctly classified training examples on the loss. On incorrect classifications, the loss is multiplied by (1 − α_*i*_), and on correct classifications, the loss is multiplied by α_*i*_. Hence, if we were to apply this factor to the Cross-Entropy loss of a single example we would get:


(3) $$\begin{array}{l} CE\left(p_{i},y_{i},\alpha_{i}\right)=\{\begin{array}{ll} -\alpha_{i}\log\left(p_{i}\right) & \text{if }y_{i}=1\\ -\left(1-\alpha_{i}\right)\log\left(1-p_{i}\right) & \text{if }y_{i}\neq 1 \end{array} \end{array}$$

Similarly, to smoothly decrease the loss on correct classifications this loss is then multiplied by ${\left(1-p_{i}\right)}^{\gamma }$, and on incorrect classifications the loss is multiplied by $p_{i}^{\gamma }$, to which we end up with what was used for the fine-tuning stage, that is, the α-balanced variant of the focal loss function:


(4) $$\begin{array}{l} FL\left(p_{i},y_{i},\alpha_{i}\right)=\{\begin{array}{ll} -\alpha_{i}{\left(1-p_{i}\right)}^{\gamma }\log\left(p_{i}\right) & \text{if }y_{i}=1\\ -\left(1-\alpha_{i}\right)p_{i}^{\gamma }\log\left(1-p_{i}\right) & \text{if }y_{i}\neq 1 \end{array} \end{array}$$

For our experiments, we chose γ = 2 and $\alpha_{i}=\frac{S_{tot}}{C\times S_{i}}$, where *S*_*tot*_ is the total number of samples in the training dataset, and *S*_*i*_ is the number of samples present in the given class.

**Figure 1:**
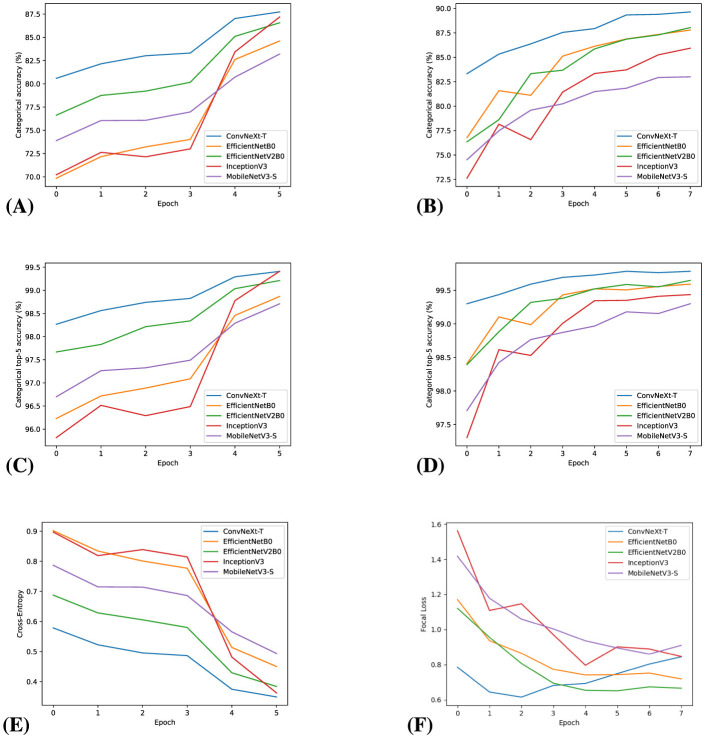
Top-1 accuracy, top-5 accuracy, and loss during fine-tuning for every epoch: 1^*st*^ column, **(A, C, E)** During initial training: 2^*nd*^ column **(B, D, F)**.

In the initial training phase, each model was initialized with weights pre-trained on the ImageNet dataset. In the first four epochs, we trained the last four layers, which correspond to the additional layers added as described in Section 2.2. To increase model accuracy, we then trained the entire model for two more epochs. Optimization hyper-parameters and algorithms for all training stages are listed in Table 1. To monitor each model's performance during training, we collected on each batch its Categorical Cross-Entropy (CE), top-1, and top-5 categorical accuracy. Such metrics were then plotted in Figure 1.

**Table 1:** Optimization parameters during each training phase.

**Parameters**	**Training phase**		
	**First 4 epochs**	**Next 2 epochs**	**Fine tuning**
Optimizer	Adam	SGD	Adam
Batch size	32	32	64
Learning rate	0.001	0.0001	–
β_1_	0.9	–	0.999
β_2_	0.999	–	0.999
Momentum	–	0.9	–
Schedule	–	–	Exponential decay
Initial learning rate	–	–	0.0001
Decay rate	–	–	0.8

The fine-tuning training stage was done in the same environment as the initial training, and the optimization hyper-parameters are listed in Table 1. Due to the higher number of training steps, we have chosen to only optimize the last third of each model to reduce computational costs. We used the same data augmentation techniques as in the previous training stage, both for the training dataset and the validation dataset.

To ensure reproducibility, the source code and datasets for the training and evaluation process have been made publicly available at https://github.com/himalayo/gravel-classification.

## 3 Results and discussion

**Figure 2:**
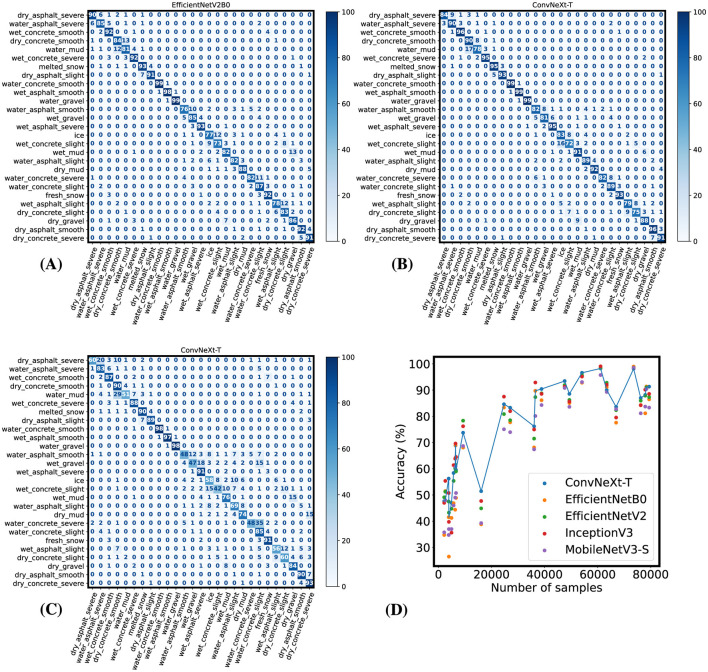
Confusion matrices of the best-performing models when evaluated on the test dataset. **(A)** Confusion matrix from evaluating the EfficientNetV2-B0 model after the fine-tuning phase. **(B)** Confusion matrix from evaluating the ConvNeXt-T model after the fine-tuning phase. **(C)** Confusion matrix for the ConvNeXt-T model after initial training. **(D)** Performance of the models after the initial training phase depending on the number of samples for each class in the dataset.

As shown in Table 2, the resulting models have a high top-5 accuracy. To further inspect its performance in each class, we have calculated the confusion matrix on every model. Since the test dataset is uneven, we have normalized the confusion matrix based on the number of samples present in the test dataset for each class (Figure 2C).

**Table 2:** Classification results on the overall test dataset before and after the fine-tuning phase, and average inference time in seconds.

**Model**	**Before**			**After**			**Inference time**
	**CE**	**Top-1**	**Top-5**	**FL**	**Top-1**	**Top-5**	
EfficientNetB0	0.65	77.29%	97.7%	0.31	89.4%	99.7%	0.49
EfficientNetV2B0	0.56	80.1%	98.4%	0.32	88.9%	99.7%	0.46
MobileNetV3-S	0.71	75.5%	97.4%	0.45	84.6%	99.4%	0.08
InceptionV3	0.52	81.2%	98.7%	0.35	87.8%	99.6%	0.96
ConvNeXt-T	0.51	81.73%	98.8%	0.26	91%	99.8%	7.50

As shown in the resulting confusion matrix for the best model in terms of top-1 categorical accuracy, its performance is greatly impacted by the subtle differences between each sub-category of a given material. This could be explained by the proportion between each sub-category in the dataset. To analyze this possibility, we plotted a graph consisting of the top-1 accuracy of each category on the *Y*-axis and the number of samples for each category on the *X*-axis (Figure 2D). As we can see, most classes that contribute to lower performance on the model have <10 thousand training samples each.

As shown in Figure 1B, there was a significant improvement of the top-1 accuracy for each model in the validation dataset after fine-tuning, with some models reaching close to 90% accuracy. This is confirmed in Figure 1F, as the resulting focal loss for every model was lower than 1, indicating significant improvement over the categories that were lowering the model's accuracy in the last training phase.

After training, we evaluated each model's performance on the test dataset, as shown in Table 2.

As indicated by the validation accuracy data collected during training, most models reached close to 90% top-1 accuracy. To further inspect the improvements on individual classes done by the fine-tuning procedures, we have calculated the confusion matrix for EfficientNetV2-B0 (Figure 2A), as it was the model with the lowest validation loss during training and the confusion matrix for the ConvNeXt-T model, for comparison with the previous training phase. Similarly to Figure 2C, these confusion matrices were normalized based on the number of samples for each class on the test dataset.

As indicated by the higher top-1 accuracy in Table 2, the fine-tuning training phase greatly improved the model's performance in every class in the ConvNeXt-T model. This is shown in Figure 2B, where lower values on the main diagonal are mostly related to miss-classification within the same material. More specifically, after the initial training phase ~18% of the images labeled as *wet gravel* were classified as *wet asphalt with severe damage*, but after fine-tuning the model with a focal loss function, the accuracy when classifying *wet gravel* increased from 47% to 81% and the percentage of this miss-classification was reduced to 6%. Similarly, the percentage of miss-classifications of *wet gravel* as *concrete road with a water puddle* was reduced from 15% to 4%, and miss-classifications of *wet gravel* as *ice* or *wet concrete with slight damage* were reduced to ~0%. The average accuracy in classifying gravel classes improved from ~76% in the ConvNeXt-T model after the initial training phase to ~89% after correcting for class imbalance, and ~90% in the EfficientNetV2-B0 model. This increase in accuracy when classifying gravel after using a focal loss function in subsequent training can be attributed to the effect of class imbalance shown in Figure 2D, where classes that are underrepresented in the training dataset show relatively low accuracy in all models.

Similarly to the results achieved by the ConvNeXt-T model, our proposed EfficientNetV2-B0 model adequately classifies all classes. We can also observe in Figure 2A that similarly to the results obtained by the ConvNeXt-T, most miss-classification cases occurred within the same material class, and with similar significance to rider's safety.

To measure every model's performance when classifying gravel under a limited resource environment, we measure inference time on every gravel class in the test dataset on a Raspberry Pi 4 Model B, and calculated the resulting average.

From the results presented in Table 2, we can observe that the fastest model in terms of inference time was MobileNetV3-S, with an average of 0.08 seconds per frame. On the other hand, the EfficientNetV2 can perform inferences ~15 times faster than the ConvNeXt-T model despite having similar top-1 accuracy.

## 4 Conclusion and future work

This research presents significant advancements in the deployment of deep learning models on embedded systems for enhancing the safety features of electric motorcycles in urban settings, specifically in detecting hazardous conditions like gravel on roads. Through comprehensive testing and evaluation, we identified EfficientNetV2 as the superior model, demonstrating an optimal trade-off between inference time and performance accuracy on the Raspberry Pi. This model's capability to deliver high computational efficiency alongside robust performance underscores its suitability for real-time applications in safety-critical environments.

Moreover, our findings also highlighted the impressive capabilities of the ConvNeXt-T model, which achieved the highest top-1 accuracy among the models tested. This underscores its potential for scenarios where maximum predictive accuracy is paramount, despite its relatively higher computational demands compared to EfficientNetV2.

An important aspect of our methodology was addressing class imbalance within the training dataset through targeted adjustments in model training approaches. This not only improved the overall accuracy of the models but also proved vital in enhancing their reliability in detecting gravel, a key concern for urban motorcycle safety.

The implications of this study are twofold. Firstly, it confirms the viability of using advanced deep-learning models on low-power devices without compromising essential performance metrics. Secondly, it provides a methodological framework for further research into AI-driven safety enhancements in the burgeoning field of intelligent and sustainable urban mobility.

Looking ahead, the integration of these AI models into actual urban transport systems will be crucial in shaping future strategies for urban mobility. The practical application of our findings can facilitate the development of more robust and scalable intelligent transport solutions, essential for addressing the growing demands of urban environments. This research not only pushes the boundaries of what is technologically possible within the constraints of low-power computing but also sets the stage for future collaborative efforts that could transform urban transportation infrastructures globally. Engaging with these challenges and opportunities will be critical as we strive to enhance the efficacy and safety of urban mobility through continued innovation in AI.

To promote transparency and facilitate reproducibility of our findings, we have made the source code, datasets, and detailed instructions publicly available in a GitHub repository. This resource is intended to support further research and the development of innovative AI-driven safety solutions for urban mobility. The repository can be accessed at https://github.com/himalayo/gravel-classification.

## Data Availability

The original contributions presented in the study are included in the article/supplementary material, further inquiries can be directed to the corresponding author.
